# Developing a Shared Language to Describe the Age-Friendly Ecosystem

**DOI:** 10.1093/geroni/igab046.1887

**Published:** 2021-12-17

**Authors:** Kim Dash, Jody Shue, Tim Driver, Alice Bonner, Leslie Pelton, Rani Snyder, Terry Fulmer

**Affiliations:** 1 Education Development Center, Waltham, Massachusetts, United States; 2 Age Friendly Foundation, Waltham, Massachusetts, United States; 3 Institute for Healthcare Improvement, Boston, Massachusetts, United States; 4 The John A. Hartford Foundation, New York, New York, United States; 5 The John A. Hartford Foundation, New York City, New York, United States

## Abstract

While multiple sectors—cities and communities, education, employment, health, and public health—have identified and implemented strategies to promote age-friendly systems, their efforts have mainly advanced in siloes. Each sector has met goals specific to its constituents, however, the major transformations required to realize systemic inclusivity and well-being among diverse groups of older adults remains indefinable. To begin to address this gap, we have engaged age-friendly sectors in a process of coordinated planning to define and operationalize an age-friendly ecosystem (AFE) that advances cross-sector and age-friendly solutions to meet the needs of all older adults. Our process borrows from Kania and Kramer (2011) who describe conditions to achieve substantial collective impact when coordinating efforts across sectors: a common agenda, shared measurement systems, mutually reinforcing activities, and continuous communication. In this presentation, we describe our stepwise process to set a common agenda, by engaging older adults and working with experts across sectors, to agree on a series of characteristics that define an AFE. Specifically, we surveyed older adults about their perceptions of an age-friendly ecosystem as well as conducted a review and analysis of relevant activities (i.e., policies, programs, and practices) associated with five age-friendly sectors. Next, activities were organized by common and defining characteristics. We then convened more than 40 international experts representing diverse age-friendly sectors to review and revise the AFE characteristics. Through structured and facilitated group processes, we worked with experts to identify and define six critical AFE characteristics as well as examples of corresponding activities.

